# Internally displaced persons and their mental health status

**DOI:** 10.1192/j.eurpsy.2021.1088

**Published:** 2021-08-13

**Authors:** N. Maruta, T. Panko, V. Fedchenko, O. Semikina

**Affiliations:** Borderline Psychiatry, “Institute of Neurology, Psychiatry and Narcology of NAMS of Ukraine” SI, Kharkiv, Ukraine

**Keywords:** Internally displaced persons, psychoeducational program

## Abstract

**Introduction:**

There are about 1.5 million internally displaced persons (IDPs) in Ukraine, which requires an assessment of their mental health.

**Objectives:**

To develop a psychoeducational program aimed at informing about the clinical manifestations (markers of symptoms) of mental disorders, the possibilities of preventing their formation and options for action in conditions of the formation or exacerbation of a mental state.

**Methods:**

270 IDPs were examined. Methods: clinical-psychopathological, psychometric, statistical.

**Results:**

Evaluation of the mental state of IDPs with symptoms of mental disorders (risk group (31.92%)) indicates the presence of various emotional disorders that formed individual syndromes – asthenic (41.18%), agrypnic (45.59%), somato-vegetative (30.88%), anxiety-depressive (20, 59%). The risk factors for the development of mental disorders in IDPs were identified - the older age is from 50 to 59 and the average age is from 40 to 49 years; lack of a complete family, lack of work, low level of social employment, lack of satisfactory living conditions, a significant decrease in the level of well-being, the preservation of the significance of factors of mental trauma, the presence of certain prenosological syndromes. The proposed psychoeducational program is built on the principle of thematic seminars with elements of social and psychological training.

**Conclusions:**

The implementation of the program provides a comprehensive impact on the cognitive, emotional, psychophysiological, behavioral and social aspects of personality functioning.

